# Supplementary material for: Inheritance bias of deletion-harbouring mtDNA in yeast: The role of copy number and intracellular selection
Source: PLoS Genet. 2025 Jun 24;21(6):e1011737. doi: 10.1371/journal.pgen.1011737 (PMC12186888; doi:10.1371/journal.pgen.1011737)
Supplement: S1 Table — The coordinates were deduced from short-read sequencing assemblies and, for some strains, verified by PCR and Sanger sequencing. (DOCX) [file pgen.1011737.s001.docx]

### Table S1. Deletion coordinates of *rho^−^* strains. The coordinates were deduced from short-read sequencing assemblies and, for some strains, verified by PCR and Sanger sequencing.

| **Strain N** | **Coordinates of remaining mtDNA (start-end, b.p.)^#^** | **Primers used for deletion position verification/comments** | **Suppressivity (mean ± SD)** |
| --- | --- | --- | --- |
| *HS rho^−^* | **31972-34167** | Isolated and characterised previously [(Karavaeva et al. 2017)](https://paperpile.com/c/Xtvm7F/Jry9) | 99.24 ± 0.42 |
| *rho^−^ 2* | **79139-11106** | Forward: 5’-CCTGCGATTAAGGCATGATGA-3’  Reverse: 5’-GAATTTCGGTGATTGGAACC-3’ | 47.61 ± 4.30 |
| *rho^−^ 4* | **81404-35820** | Forward: 5’-CCTGCGATTAAGGCATGATGA-3’  Reverse 5’-ATGATAGATATCTGGGGTCC-3’ | 50.38 ± 5.96 |
| *rho^−^ 5* | 84230-34944 | Forward: 5’-GGATCAAACATTACCCGTTG-3’  Reverse: 5’-CATGACCCTAAAATGTTAACC-3’  PCR produced multiple products (250 bp and 2500 bp). Exact deletion coordinates were not verified and taken from illumina sequencing assembly. | 90.21 ± 5.14 |
| *rho^−^ 6* | **79139-11106** | Forward: 5’-CCTGCGATTAAGGCATGATGA-3’  Reverse: 5’-GAATTTCGGTGATTGGAACC-3’ | 68.41 ± 9.64 |
| *rho^−^ 9* | 28455-6193 |  | 68.76 ± 3.38 |
| *rho^−^ 10* | 34762-56343 |  | 76.64 ± 2.70 |
| *rho^−^ 11* | 22690-35669 |  | 88.10 ± 8.98 |
| *rho^−^ 12* | 13391-35721 |  | 89.17 ± 2.66 |
| *rho^−^ 13* | 6205-52357 |  | 44.95 ± 10.95 |
| *rho^−^ 14* | **6987-52813** | Forward: 5’-GGAAATATAAAAACCGAAGG-3’  Reverse: 5’-CTGCTGGCACAAATATTAGTC-3’ | 74.44 ± 15.79 |
| *rho^−^ 15* | 30305-82529 |  | 74.41 ± 6.18 |
| *rho^−^ 18* | 29349-9065 |  | 53.93 ± 12.15 |
| *rho^−^ 19* | 71153-34184 |  | 82.06 ± 7.38 |
| *rho^−^ 20* | 73258-34273 |  | 87.15 ± 5.55 |
| *rho^−^ 21* | **35103-55705** | Forward: 5’-CTGCAATATCTTTTGCATTTG-3’  Reverse: 5’-GATGTCGTAACCATTAGACG-3’ | 28.06 ± 9.50 |
| *rho^−^ 22* | 70617-12320 |  | 70.77 ± 0.68 |
| *rho^−^ 45* | 53915-82908 |  | 77.80 ± 5.55 |
| *rho^−^ Ia14* | 41776-66304 |  | 43.85 ± 9.75 |
| *rho^−^ Ib28* | 28052-67035 |  | 45.18 ± 17.20 |
| *rho^−^ IIa3* | 41659-4171 |  | 44.92 ± 13.38 |
| *rho^−^ IIa10* | 8626-28624 |  | 19.37 ± 8.06 |
| *rho^0^ Ib24* | n.d. appeared to be *rho^0^* |  | 22.14 ± 16.01 |
| *rho^−^ IIc11* | 29409-4169 |  | 63.11 ± 3.41 |

^#^ coordinates according to the S288C reference genome annotation.
