# Supplementary material for: Inheritance bias of deletion-harbouring mtDNA in yeast: The role of copy number and intracellular selection
Source: PLoS Genet. 2025 Jun 24;21(6):e1011737. doi: 10.1371/journal.pgen.1011737 (PMC12186888; doi:10.1371/journal.pgen.1011737)
Supplement: S2 Table — Trim mean and median values were calculated for the entire mitochondrial genomes. In the rest of the columns, the average read depth was calculated in specified positions. (DOCX) [file pgen.1011737.s002.docx]

### Table S2. Quantification of mtDNA copy number from NGS data: depth of reads mapped to mtDNA normalised to depth of reads mapped to the nuclear genome. Trim mean and median values were calculated for the entire mitochondrial genomes. In the rest of the columns, the average read depth was calculated in specified positions.

| **Strain** | **Mean**  **(trim = 0.1)** | **Median** | **Mean,**  **primer set 1 position (31222-31305)** | **Mean,**  **primer set 2 position**  **(8002-8153)** | **Mean,**  **primer set 3 position (48195-48296)** | **Mean,**  **GC rich region (14100-21100)** |
| --- | --- | --- | --- | --- | --- | --- |
| *rho^+^ strain* | 4.37 | 3.00 | 6.56 | 4.51 | 4.81 | 15.45 |
| *HS rho*^−^ | 40.13 | 0.04 | 679.69 | NA | NA | NA |
| *rho*^−^ *2* | 10.16 | 9.96 | NA | 24.10 | NA | 0.03 |
| *rho*^−^ *4* | 9.94 | 5.08 | 87.76 | 6.46 | NA | 30.82 |
| *rho*^−^ *5* | 2.00 | 1.32 | 183.35 | 0.97 | NA | 2.44 |
| *rho*^−^ *6* | 11.55 | 6.11 | 46.19 | 0.08 | 33.04 | 0.47 |
| *rho*^−^ *9* | 10.08 | 8.34 | 41.38 | NA | 14.70 | 0.03 |
| *rho*^−^ *10* | 7.59 | 6.50 | NA | NA | 12.72 | 0.01 |
| *rho*^−^ *11* | 16.29 | 0.75 | 235.83 | NA | NA | 0.07 |
| *rho*^−^ *12* | 2.79 | 0.89 | 68.96 | NA | NA | 0.83 |
| *rho*^−^ *13* | 8.46 | 4.51 | 10.32 | 3.72 | 3.85 | 24.28 |
| *rho*^−^ *14* | 4.81 | 2.95 | 10.97 | 35.33 | 4.56 | 2.57 |
| *rho*^−^ *15* | 5.45 | 3.92 | 28.55 | 0.04 | 5.32 | 0.04 |
| *rho*^−^ *18* | 10.63 | 8.06 | 27.52 | 3.62 | 21.43 | 0.14 |
| *rho*^−^ *19* | 6.60 | 3.52 | 821.52 | 0.97 | NA | 13.10 |
| *rho*^−^ *20* | 2.36 | 1.22 | 187.83 | 0.53 | NA | 4.79 |
| *rho*^−^ *21* | 7.76 | 5.41 | 0.10 | NA | 13.11 | 0.04 |
| *rho*^−^ *22* | 46.62 | 45.09 | 0.21 | 130.47 | 0.40 | 0.21 |
| *rho*^−^ *45* | 20.61 | 19.34 | 0.50 | NA | NA | 0.27 |
| *rho^−^ Ia14* | 7.66 | 6.90 | NA | NA | 21.45 | NA |
| *rho^−^ Ib28* | 10.63 | 9.98 | 9.85 | NA | 19.35 | NA |
| *rho^−^ IIa3* | 10.77 | 9.25 | 28.64 | NA | NA | 25.60 |
| *rho^−^ IIa10* | 12.15 | 10.12 | NA | NA | 25.72 | NA |
| *rho^−^ IIc11* | 59.13 | 53.35 | NA | 0.06 | 55.06 | NA |
| *rho^0^ strain 1* | 0.19 | 0.19 | 0.23 | NA | NA | 0.20 |
| *rho^0^ strain 2* | 0.08 | 0.07 | NA | NA | NA | NA |
