# Supplementary material for: Inheritance bias of deletion-harbouring mtDNA in yeast: The role of copy number and intracellular selection
Source: PLoS Genet. 2025 Jun 24;21(6):e1011737. doi: 10.1371/journal.pgen.1011737 (PMC12186888; doi:10.1371/journal.pgen.1011737)
Supplement: S3 Table — (DOCX) [file pgen.1011737.s003.docx]

### Table S3. Primers used for quantitative PCR (qPCR) of mtDNA to nDNA ratio.

| **Name** | **Sequence** | **Product, b.p.** | **Annealing temperature** |
| --- | --- | --- | --- |
| qPCR region 1 | Forward: att-cca-cct-tca-gcg-tag-t  Reverse: ggt-tcg-gtc-ctc-cct-tac | 83 | 60°C |
| qPCR region 2 | Forward: ttc-gca-cta-atc-act-cat-cac  Reverse: ccc-tac-ggt-aac-tgt-att-tca-ac | 152 | 60°C |
| qPCR region 3 | Forward: gta-tta-tta-cgg-atg-atg-tag-gat  Reverse: aag-gat-ggt-tga-ctg-agt | 98 | 53°C |
| ACT1 | Forward: tcc-cag-gta-ttg-ccg-aaa-gaa-tgc  Reverse: gcc-aag-ata-gaa-cca-cca-atc-cag-ac | 124 | 58°C |
