# Supplementary material for: Inheritance bias of deletion-harbouring mtDNA in yeast: The role of copy number and intracellular selection
Source: PLoS Genet. 2025 Jun 24;21(6):e1011737. doi: 10.1371/journal.pgen.1011737 (PMC12186888; doi:10.1371/journal.pgen.1011737)
Supplement: S4 Table — The mtDNA copy number was determined as the ratio of mitochondrial to nuclear genome loci abundance, calculated as -logCt. The positions of the amplified regions 1 and 3 used for the mtDNA quantification are illustrated in S1 Fig. (DOCX) [file pgen.1011737.s004.docx]

### Table S4. Quantification of mtDNA copy number using qPCR. The mtDNA copy number was determined as the ratio of mitochondrial to nuclear genome loci abundance, calculated as -logCt. The positions of the amplified regions 1 and 3 used for the mtDNA quantification are illustrated in Figure S1.

| **Strain** | **mtDNA/**  **nDNA** | **mtDNA/nDNA**  **(region 1), mean** | **mtDNA/nDNA**  **(region 1),**  **SD** | **mtDNA/nDNA**  **(region 3), mean** | **mtDNA/nDNA**  **(region 3),**  **SD** | **mtDNA *rho^−^* normalised to WT** |
| --- | --- | --- | --- | --- | --- | --- |
| *rho^+^* | 24 | 22 | 4.4 | 26.6 | 9.4 | 1 |
| *HS rho*^−^ | 686 | 686 | 216 | NA | NA | 28.3 |
| *rho*^−^ *4* | 206 | 206 | 84 | NA | NA | 8.5 |
| *rho*^−^ *5* | 357 | 357 | 99 | NA | NA | 14.7 |
| *rho*^−^ *6* | 62 | 70 | 45 | 56 | 16 | 2.6 |
| *rho*^−^ *9* | 55 | 63 | 34 | 48 | 24 | 2.3 |
| *rho*^−^ *10* | 107 | NA | NA | 108 | 25 | 4.4 |
| *rho*^−^ *11* | 341 | 341 | 129 | NA | NA | 14.1 |
| *rho*^−^ *12* | 197 | 197 | 77 | NA | NA | 8.1 |
| *rho*^−^ *13* | 50 | 49 | 17.5 | 52 | 26 | 2.1 |
| *rho*^−^ *14* | 34 | 48 | 16.4 | 24.5 | 10 | 1.4 |
| *rho*^−^ *15* | 68 | 102 | 58 | 46 | 11 | 2.8 |
| *rho*^−^ *18* | 42 | 41 | 7.9 | 43 | 12 | 1.7 |
| *rho*^−^ *19* | 412 | 412 | 106 | NA | NA | 17.0 |
| *rho*^−^ *20* | 233 | 232 | 57 | NA | NA | 9.6 |
| *rho*^−^ *21* | 124 | NA | NA | 124 | 59 | 5.1 |
