# Supplementary material for: Inheritance bias of deletion-harbouring mtDNA in yeast: The role of copy number and intracellular selection
Source: PLoS Genet. 2025 Jun 24;21(6):e1011737. doi: 10.1371/journal.pgen.1011737 (PMC12186888; doi:10.1371/journal.pgen.1011737)
Supplement: S1 Fig — (PDF) [file pgen.1011737.s006.pdf]

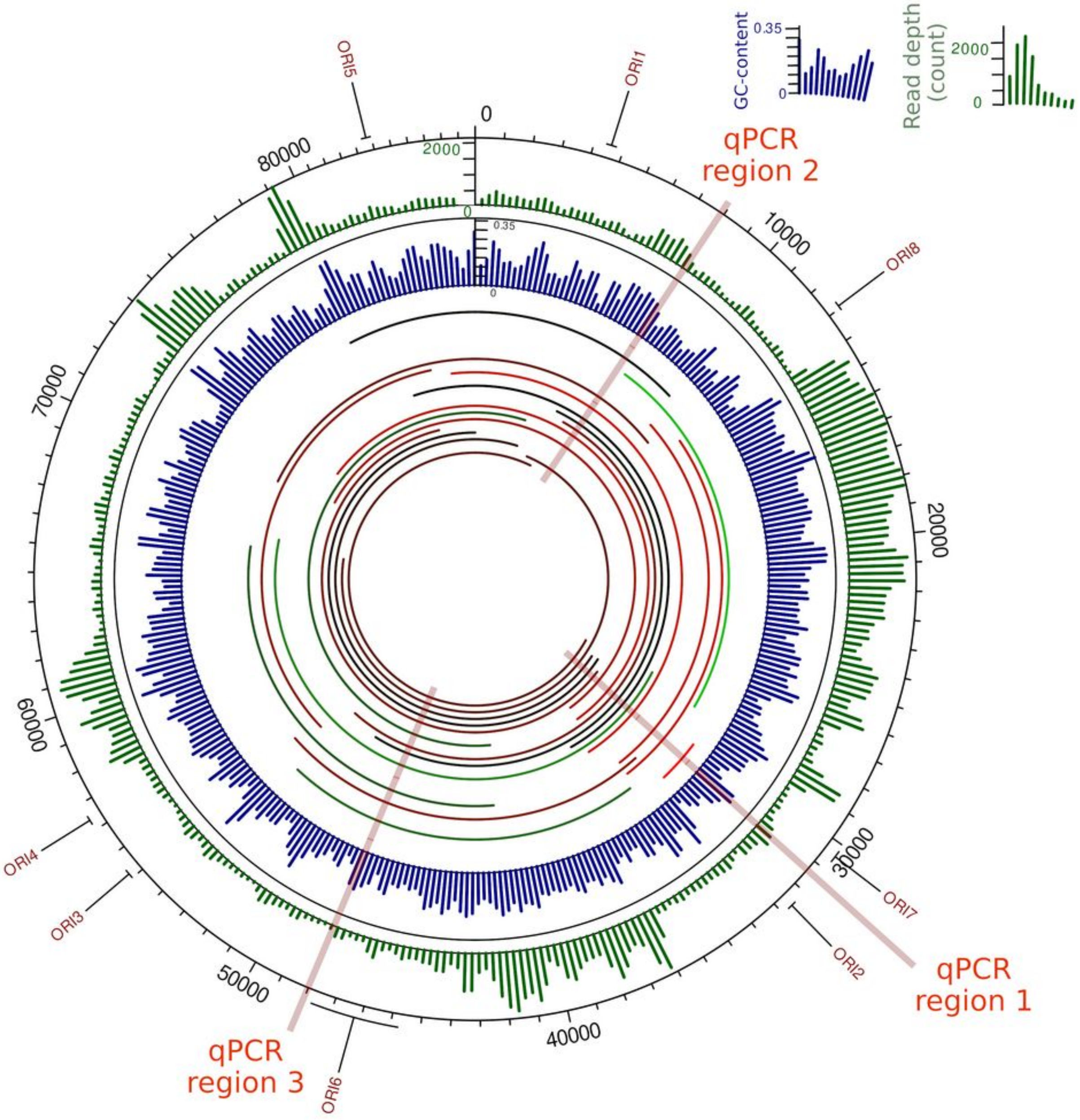

Figure S1. Relative positions of the regions used for mtDNA quantitative PCR and the regions retained in *rho*⁻ mitochondrial genomes. The arcs on the map illustrate the mtDNA segments preserved in the spontaneous *rho*⁻ mutants examined in this study. Read depth is shown for wild-type *rho*⁺ mtDNA.
