# Supplementary material for: Inheritance bias of deletion-harbouring mtDNA in yeast: The role of copy number and intracellular selection
Source: PLoS Genet. 2025 Jun 24;21(6):e1011737. doi: 10.1371/journal.pgen.1011737 (PMC12186888; doi:10.1371/journal.pgen.1011737)
Supplement: S3 Fig — (PDF) [file pgen.1011737.s008.pdf]

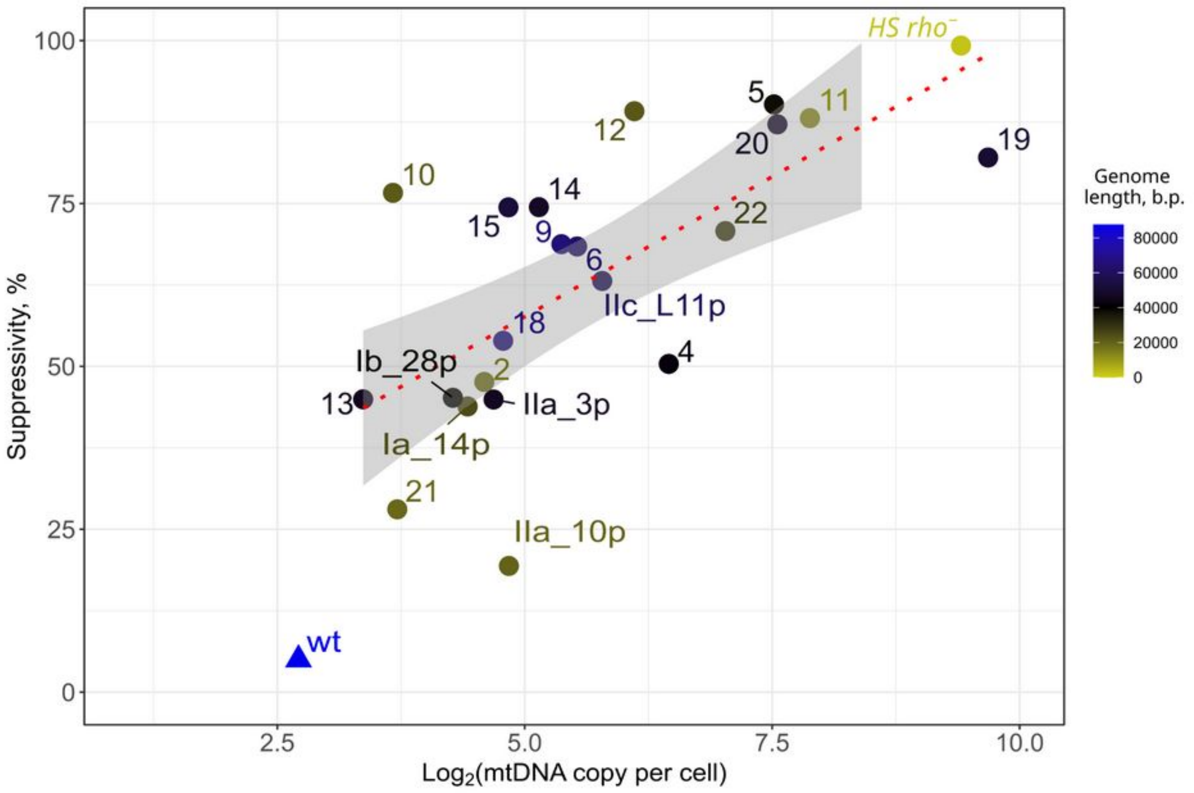

Figure S3. Correlation of *rho*⁻ mtDNA copy number per nuclear genome estimated from NGS data with suppressivity (as in Figure 1C, but with the strain names added as point labels).
