# Supplementary material for: Inheritance bias of deletion-harbouring mtDNA in yeast: The role of copy number and intracellular selection
Source: PLoS Genet. 2025 Jun 24;21(6):e1011737. doi: 10.1371/journal.pgen.1011737 (PMC12186888; doi:10.1371/journal.pgen.1011737)
Supplement: S4 Fig — (PDF) [file pgen.1011737.s009.pdf]

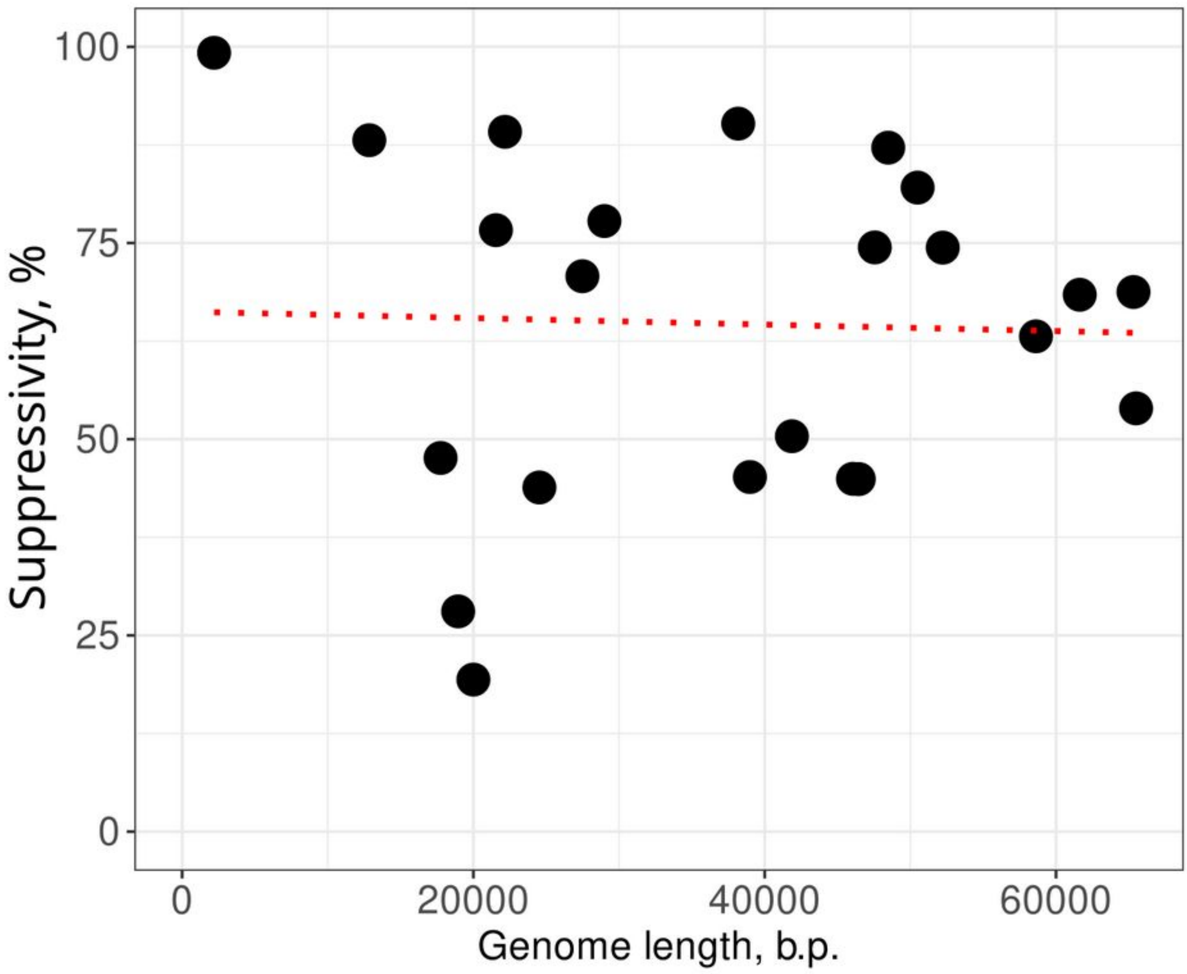

Figure S4. No apparent correlation between the genome lengths of *rho*⁻ mtDNA and their suppressivity.
