# Supplementary material for: Inheritance bias of deletion-harbouring mtDNA in yeast: The role of copy number and intracellular selection
Source: PLoS Genet. 2025 Jun 24;21(6):e1011737. doi: 10.1371/journal.pgen.1011737 (PMC12186888; doi:10.1371/journal.pgen.1011737)
Supplement: S5 Fig — (PDF) [file pgen.1011737.s010.pdf]

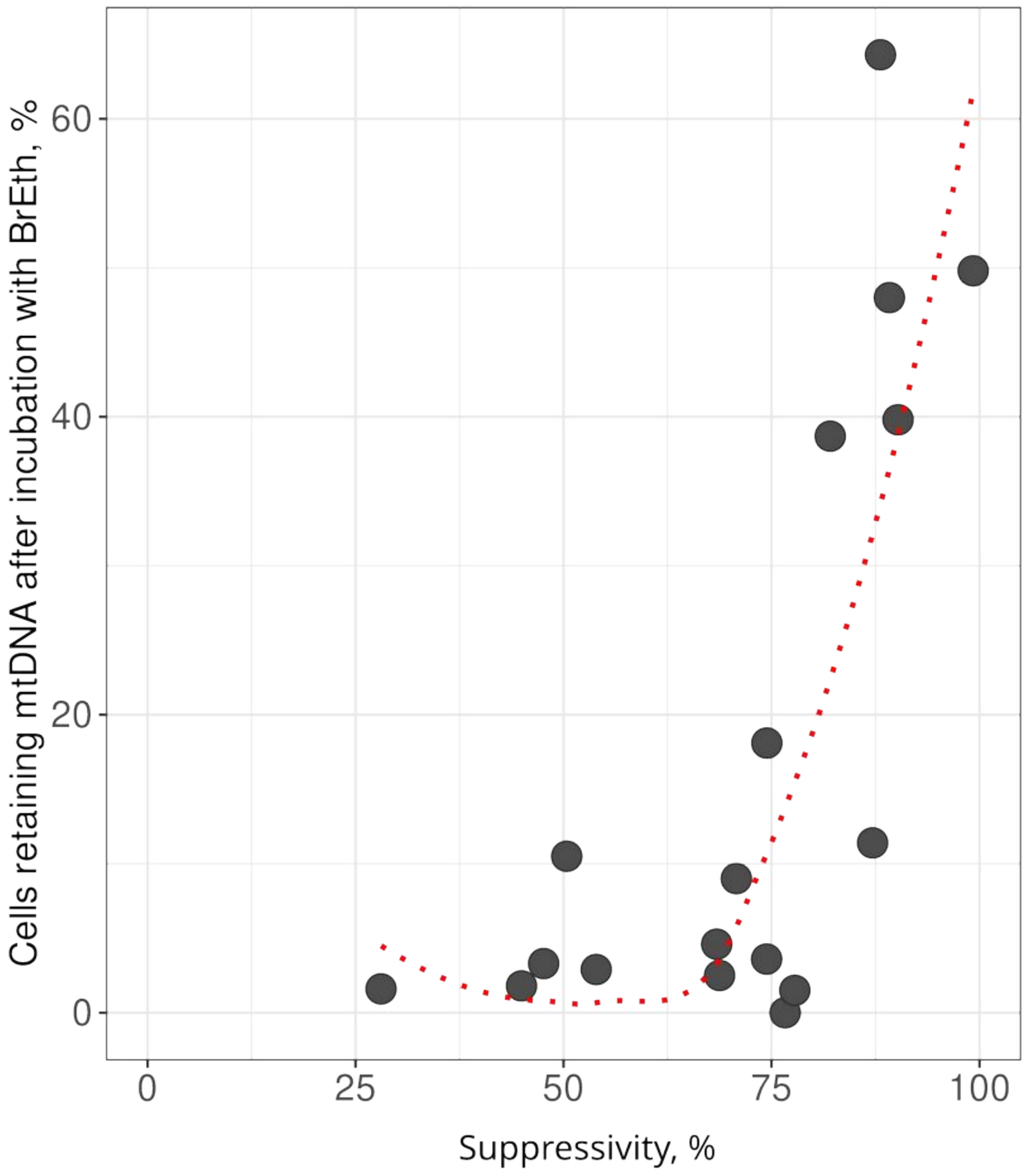

Figure S5. The ability of *rho*⁻ yeast cells to retain mtDNA upon growth with the DNA-intercalating agent ethidium bromide correlates with the *rho*⁻ strain's suppressivity (Kendall's rank correlation tau = 0.56, p-value = $8*10^{-4}$)
