# Supplementary material for: Inheritance bias of deletion-harbouring mtDNA in yeast: The role of copy number and intracellular selection
Source: PLoS Genet. 2025 Jun 24;21(6):e1011737. doi: 10.1371/journal.pgen.1011737 (PMC12186888; doi:10.1371/journal.pgen.1011737)
Supplement: S6 Fig — (A) Schematic showing the experimental setup for measuring the suppressivity drift over generations; (B) Change in suppressivity between the first (green circles) and subsequent (orange and blue circles) assessments (n = 76). The orange vs blue color of the circle illustrates the direction of change in subsequent experiments compared to the first one, with orange indicating a decrease and blue indicating an increase of suppressivity over generations. X labels the results of individual experiments. (PDF) [file pgen.1011737.s011.pdf]

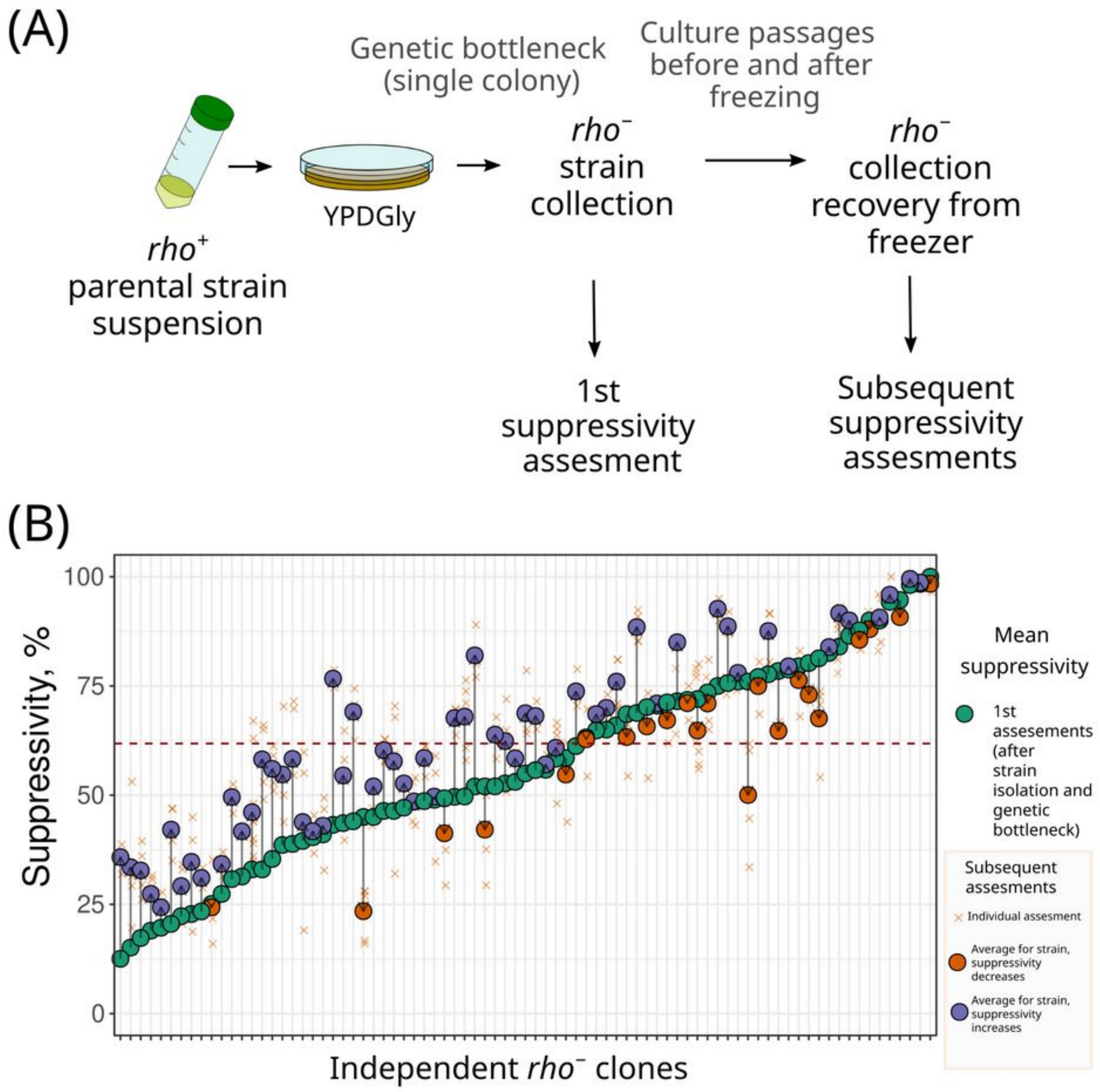


Figure S6. Suppressivity increases within yeast subclones. (A) The experimental setup for measuring the suppressivity drift over generations; (B) Change in suppressivity between the first (green circles) and subsequent (orange and blue circles) assessments (n = 76). The orange vs blue color of the circle illustrates the direction of change in subsequent experiments compared to the first one, with orange indicating a decrease and blue indicating an increase of suppressivity over generations. X labels the results of individual experiments.
