# Supplementary material for: Inheritance bias of deletion-harbouring mtDNA in yeast: The role of copy number and intracellular selection
Source: PLoS Genet. 2025 Jun 24;21(6):e1011737. doi: 10.1371/journal.pgen.1011737 (PMC12186888; doi:10.1371/journal.pgen.1011737)
Supplement: S7 Fig — (A) Simulation algorithm, see details in the Methods section. (B) The simulation predicts the proportion of cells containing only rho+ mtDNA (represented by the blue area) and those with only rho− mtDNA (represented by the red area). Each plot represents an overlay of the results of ten simulations, to overlay blue and red areas that illustrate homoplasmic cells we set their opacity to 0.1. The not coloured areas correspond to the cell retaining heteroplasmy. Three plots represent simulation results with different intracellular fitness levels of rho− mtDNA (1, 1.3, and 1.6). The relative cell-level fitness of rho− cells was simulated using doubling times of 140 minutes and 190 minutes. The pathogenicity threshold was set at 0.5, indicating that a rho− cell-level phenotype only manifests when more than half of the mtDNA in a cell is rho−. (PDF) [file pgen.1011737.s012.pdf]

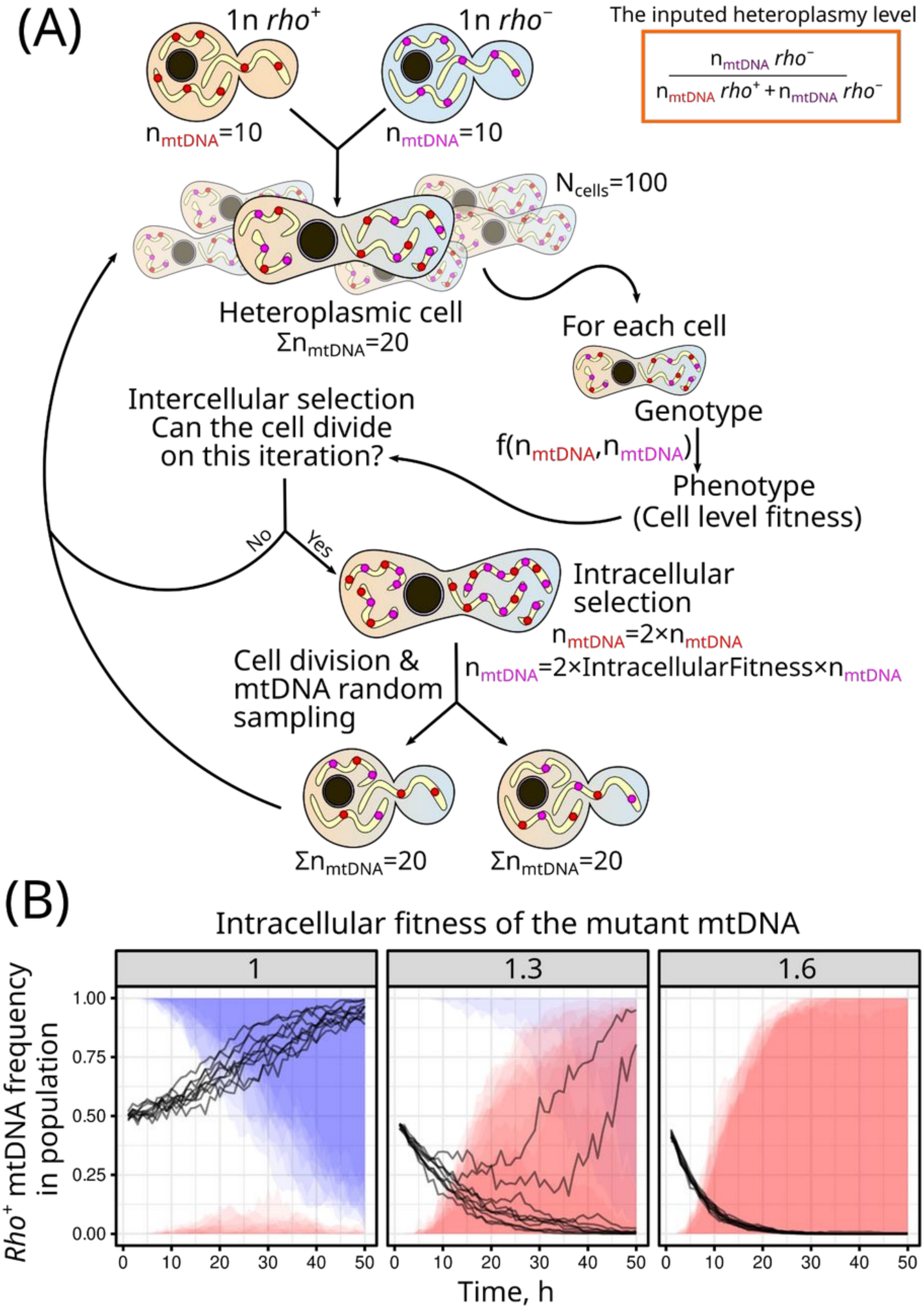
(A)
1n rho+
1n rho−
The inputed heteroplasmy level
nmtDNA rho− / (nmtDNA rho+ + nmtDNA rho−)
nmtDNA=10
nmtDNA=10
Ncells=100
Heteroplasmic cell
Σn mtDNA=20
For each cell
Genotype
f(nmtDNA,nmtDNA)
Phenotype
(Cell level fitness)
Intercellular selection
Can the cell divide
on this iteration?
No
Yes
Intracellular
selection
nmtDNA=2×nmtDNA
nmtDNA=2×IntracellularFitness×nmtDNA
Cell division &
mtDNA random
sampling
Σn mtDNA=20
Σn mtDNA=20
(B)
Intracellular fitness of the mutant mtDNA
1
1.3
1.6
Rho+ mtDNA frequency
in population
1.00
0.75
0.50
0.25
0.00
0 10 20 30 40 50
Time, h

Figure S7. mtDNA selection in heteroplasmic yeast cells: a simulation guided by experimental data. (A) Simulation algorithm, see details in the Methods section. (B) The simulation predicts the proportion of cells containing only *rho*$^+$ mtDNA (represented by the blue area) and those with only *rho*$^-$ mtDNA (represented by the red area). Each plot represents an overlay of the results of ten simulations, to overlay blue and red areas that illustrate homoplasmic cells we set their opacity to 0.1. The not coloured areas correspond to the cell retaining heteroplasmy. Three plots represent simulation results with different intracellular fitness levels of *rho*$^-$ mtDNA (1, 1.3, and 1.6). The relative cell-level fitnesses of *rho*$^+$ and *rho*$^-$ cells was simulated using doubling times of 140 minutes and 190 minutes correspondingly. The pathogenicity threshold was set at 0.5, indicating that a *rho*$^-$ cell-level phenotype only manifests when more than half of the mtDNA in a cell is *rho*$^-$.
