# Supplementary material for: Inheritance bias of deletion-harbouring mtDNA in yeast: The role of copy number and intracellular selection
Source: PLoS Genet. 2025 Jun 24;21(6):e1011737. doi: 10.1371/journal.pgen.1011737 (PMC12186888; doi:10.1371/journal.pgen.1011737)
Supplement: S8 Fig — Simulation data and experimental data plotted in the coordinates of starting heteroplasmy level ~ suppressivity (same as in the Fig 4B); Points represent simulated data, crosses represent experimental data points for the 22 rho− strains; a line connects the points obtained with the simulation with no replication advantage of rho− mtDNA (Intracellular rho− mtDNA fitness equal to 1.0). (PDF) [file pgen.1011737.s013.pdf]

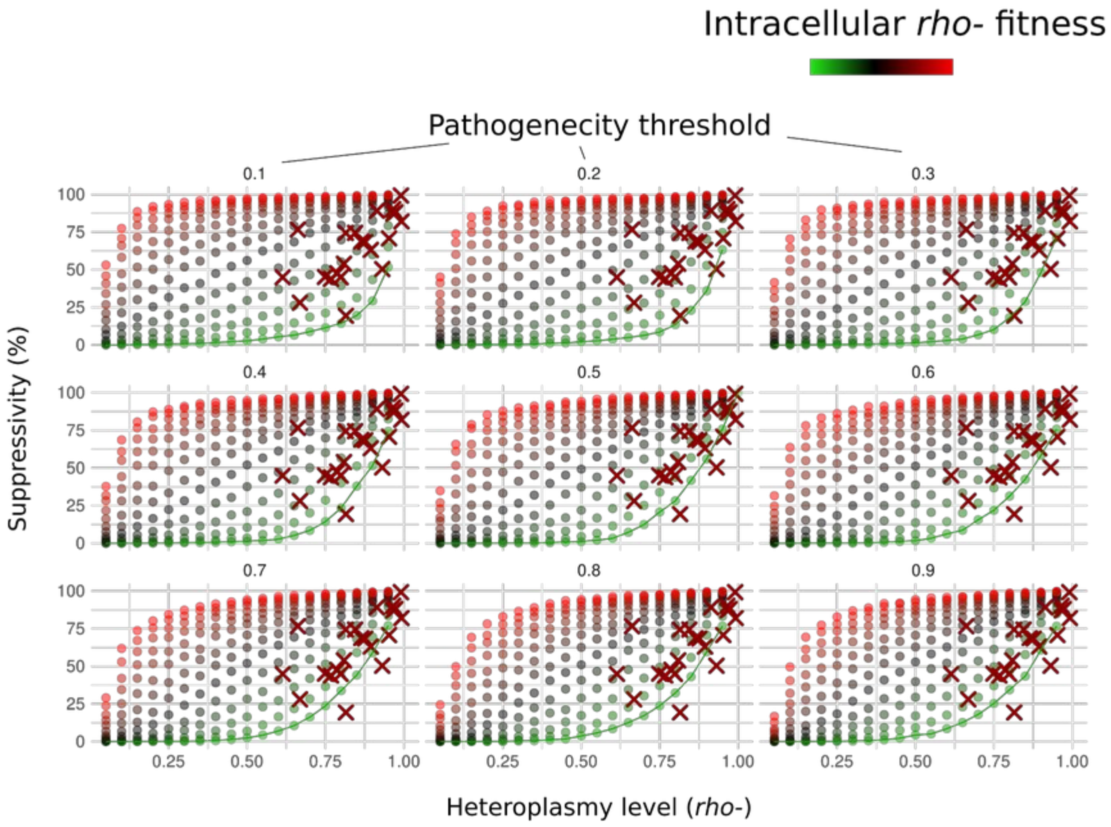

Figure S8. Variation of pathogenicity threshold in the simulations of suppressivity. Simulation data and experimental data plotted in the coordinates of starting heteroplasmy level ~ suppressivity (same as in the Figure 4B); Points represent simulated data, crosses represent experimental data points for the 22 *rho*$^{-}$ strains; a line connects the points obtained with the simulation with no replication advantage of *rho*$^{-}$ mtDNA (Intracellular *rho*$^{-}$ mtDNA fitness equal to 1.0)
