# Supplementary material for: Inheritance bias of deletion-harbouring mtDNA in yeast: The role of copy number and intracellular selection
Source: PLoS Genet. 2025 Jun 24;21(6):e1011737. doi: 10.1371/journal.pgen.1011737 (PMC12186888; doi:10.1371/journal.pgen.1011737)
Supplement: S10 Fig — Individual lines illustrate the predictions for individual rho− strains, and the red bold line shows the average value. (PDF) [file pgen.1011737.s015.pdf]

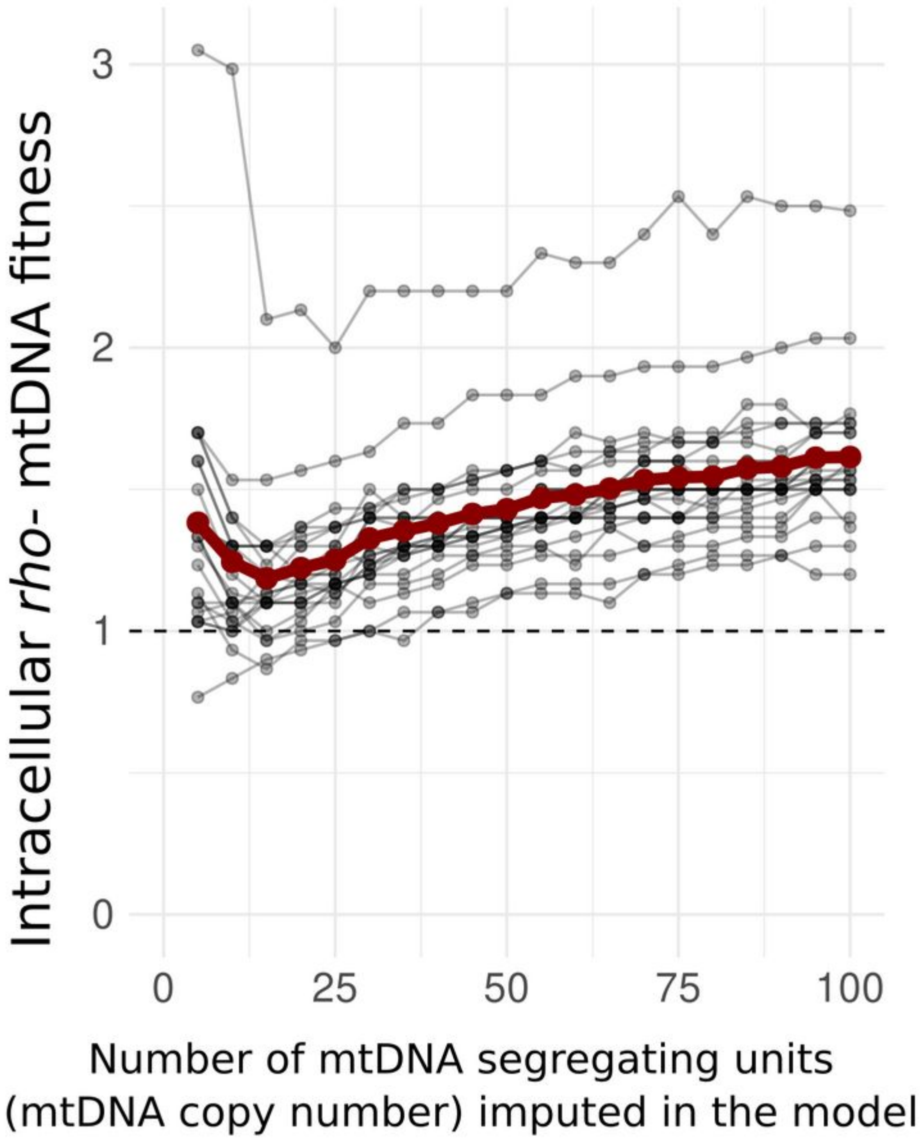

Figure S10. Relative intracellular fitness of *rho*$^{-}$ mtDNA variants calculated from the k-nearest neighbours for simulations with different mtDNA copy numbers (number of mtDNA segregating units). Individual lines illustrate the predictions for individual *rho*$^{-}$ strains, and the red bold line shows the average value.
