# Supplementary material for: Inheritance bias of deletion-harbouring mtDNA in yeast: The role of copy number and intracellular selection
Source: PLoS Genet. 2025 Jun 24;21(6):e1011737. doi: 10.1371/journal.pgen.1011737 (PMC12186888; doi:10.1371/journal.pgen.1011737)
Supplement: S11 Fig — Arrows indicate putative mitochondrial nucleoid foci in rho− cells. Images are representative and were acquired and processed independently on different days. (PDF) [file pgen.1011737.s016.pdf]

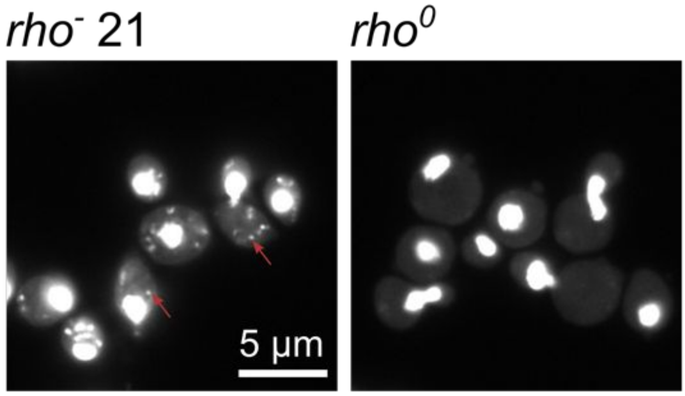

Figure S11. DAPI staining of *rho⁰* and *rho⁻* cells. Arrows indicate putative mitochondrial nucleoid foci in *rho⁻* cells. Images are representative and were acquired and processed independently on different days.
