# Supplementary material for: Inheritance bias of deletion-harbouring mtDNA in yeast: The role of copy number and intracellular selection
Source: PLoS Genet. 2025 Jun 24;21(6):e1011737. doi: 10.1371/journal.pgen.1011737 (PMC12186888; doi:10.1371/journal.pgen.1011737)
Supplement: Attachment — Submitted filename: Answers2Reviewers.docx [file pgen.1011737.s017.docx]

## Letter to the editor

Dear Editors,

Please find the revised version of our manuscript, entitled "Inheritance bias of deletion-harbouring mtDNA in yeast: the role of copy number and intracellular selection". In the revised version, we have made every effort to improve the manuscript in accordance with the reviewers' recommendations and addressed their questions. During the revision, we sequenced and analyzed additional yeast strains; the new data confirmed the presence of secondary mutations in some subclones of rho- strains. This new data has been added as Figure 2B, and the raw sequencing data have been uploaded to the SRA archive mentioned in the Data Availability statement. We have also added the missing details to the methods section and provided additional supplementary figures with technical data. We took special care to make the description of the stochastic model explicit and to clarify mtDNA copy number measurements. We have tried our best to explain the choice of assumptions and parameters. The new supplementary figures S2 and S3 were suggested by the reviewers, and new figure S11 provides an example of DAPI staining of yeast cells with and without mtDNA. Additionally, we have added supplementary Table S5 with the data underlying Figure 3. As suggested by the reviewers, we also made some minor improvements to Figures 1 and 3. Please find the manuscript with highlighted changes included with this submission.

During the revision stage, most of the experiments and analysis were performed by Georgii Muravyev; therefore, we agreed to add him as a coauthor. All authors have agreed to the new author list. In accordance with the journal guidelines, I am attaching an Author Change Form with this resubmission.

sincerely yours,

Dmitry Knorre

**Reviewers' comments:**

Reviewer's Responses to Questions

**Comments to the Authors:
Please note here if the review is uploaded as an attachment.**

## Reviewer #1:

Kashko et al illuminate the interesting question of why, following the mating of gametes with mutated and intact mtDNA respectively, mutated mtDNA take over in diploid cell populations expanded from zygotes, despite diploid cells with mutated mtDNA performing worse than diploids cells with intact mtDNA. They distinguish between two not mutually exclusive explanations: first, that mutated mtDNA reach higher copy numbers in haploid gametes, and therefore become more numerous in zygotes, and second, that mutated mtDNAs have an intracellular competitive advantage relative intact mtDNAs in diploids. They collect data on mtDNA copy number for mutated and intact mtDNA in haploids, and measure their effect on cell fitness, approximated as maximum growth rate. They also measure the capacity of mutated mtDNA to replace intact mtDNA, when gametes are hybridised to generate diploid cell populations. Finally, they explore using population genetics/dynamics simulations in what scenarios simulated cells with mutated mtDNA comes to dominate in diploid cells obtained from mating gametes with intact and mutated mtDNA respectively. Based on these simulations, they conclude that both higher copy number of mutated mtDNA in haploid gametes, and their ability to outrival intact mtDNA within diploid cells, helps mutated mtDNA take over - even if mutated mtDNA reduce the cell fitness. I very much like the paper’s combination of experiments and simulations, which surely must be the right-way forward to address this interesting problem – but I do have a number of reservations.

1. The methods section is sparse, making it hard to evaluate whether experiments and simulations are soundly designed and data can be trusted.

A) A more detailed description of nutrient and energy concentrations, pH and how the pH is maintained would be welcome, given that the petite slow growth phenotype is essentially an intracellular aminoacid deficiency, that likely is influenced by external concentrations and the protonmotive force. It is also not clear to me how cultures and precultures were done or which experiments were performed on rich and synthetic medium respectively. Likewise, the genotypes of the W303 strain used is not given.

We updated the Methods section to include strain genotypes, as well as more details on yeast cultivation. All experiments for suppressivity assessment were performed in rich media, and cells were plated onto synthetic selective media only after mating, which we clarified in the Methods section of the revised manuscript. While we admit that in our experimental conditions, pH was not controlled or maintained during strain mating (20 h at room temperature with no shaking), we presume that it was changing similarly in all experiments, including control matings with *rho^+^* and *rho^0^* strains. Moreover, as there seems to be little difference in growth rate between *rho^−^* strains (as illustrated in Figure 3), the measured suppressivity is likely to reflect the properties of specific deletions in mtDNA.

B) I am not sure how the rho0 control was obtained, or more generally what operative distinction between rho0 and rho- the authors use. Rho- cells may retain small mtDNA segments, often circularized, that NGS sequencing and qPCR fail to detect unless care is taken - making them appear like rho0. A good rho0 control would be e.g. a mip1delta strain.

*Rho^0^* strain was obtained by incubation with ethidium bromide. The *petite* phenotype (respiratory deficiency due to the absence of functional mitochondrial DNA) was confirmed by the absence of growth on a nonfermentable carbon source (YPGly). As *rho^−^* and *rho^0^* strains have the same growth phenotype, we used fluorescence microscopy to check whether the *rho^0^* candidate strains contained mitochondrial DNA that in *S. cerevisiae* appears as easily distinguishable cytoplasmic foci. We stained *rho^0^* candidate strains with DAPI and selected only the strains that lacked cytoplasmic foci. We added a more detailed description of this procedure to the Methods section “Yeast strains, growth conditions and reagents”, as well as Figure S11 with the example of DAPI staining of *rho^0^* vs *rho^−^* cells.

We admit that this way of making might not be sensitive enough to distinguish from *rho^−^* cells with very low mtDNA copy numbers; however, *rho^−^* strains usually have elevated mtDNA amounts and always carry cytoplasmic DNA foci as observed from DAPI staining. Moreover, in our system, we could further confirm that *rho^0^* cells carry no mtDNA by crossing them to a *rho^+^* strain in a suppressivity assay. As no mutant mtDNA is present, suppressivity levels in such matings do not exceed standard *rho^+^* values.

C) the number and type of replicates and how these are handled statistically are not clear to me, nor are error bars and whiskers explained in all figures.

Where possible, we have displayed individual data points alongside boxplots using default parameters from the R ggplot2 library. These individual data points represent either separate strains or measurements taken on different dates, depending on the experimental setup. By default, the boxplot borders denote the first and third quartiles, while the whiskers extend from these hinges to the largest and smallest values within 1.5 times the interquartile range (IQR). We have added this explanation to both the Data Analysis and Visualization section of the Methods and to Figure 3B, where data are presented as mean and standard deviation. We also added the number of separate day experiments (n = 4) to the Figure 3B legend.

D) I cannot find a methods section describing how the EtBr experiments and associated microscopy was performed.

We are very grateful to the reviewer for pointing it out. We added the associated Methods section under “Assessment of ethidium-bromide-induced mtDNA loss”.

E) I cannot find a description of the growth experiments and how maximum growth rates were extracted measured.

We updated the methods section to explain the assessment of growth rate in both the suspensions (Figure 3A, B) and during the suppressivity measurement experiment (Figure 3C-E). We also briefly explained how we calculated mu using a custom R script and uploaded it to GitHub: https://github.com/Knorre-lab/GrowthCurves.

I do have some concerns here. From Fig 3A, it appears that OD550, was measured while OD 600-610 is more normal. However, optical density and cell density is not linear for most spectrometers. This is normally accounted for by diluting cultures down a more linear part of the OD interval, but from the reported OD values, this doesn’t seem to have been done here. Unless this is accounted for (see e.g. PMID 12489126), the observed OD may be very misleading, as may be the extracted maximum growth rates.

We acknowledge that measuring growth rates in batch cultures (e.g., using a multiwell spectrophotometer, as in our case) has limited applicability for determining the maximum growth rate. This is because optical density (OD) is linearly correlated with cell density only within a narrow range of values, typically from 0.1 to 0.6 in our experience. However, our study employed a highly standardized protocol, consistent with our previous work (e.g., PMID: 35401495, PMID: 35331734), which allows for reliable comparison of the strains. Therefore, we suggest that our approach, combined with the extraction of growth parameters, enables us to compare growth rates effectively. Importantly, Figures 3A and B are intended to assess the differences between our *rho−* and *rho0* strains rather than determine the actual growth rate (mu).

To avoid potential misinterpretation, we have added the following clarification to the Methods section: “It should be noted that the calculated maximal growth rate (µ_max_) values may be biased, as they were measured in batch culture experiments where OD may not linearly depend on cell density in dense cultures.”

Moreover, it is not possible to tell to which extent extracted maximum growth rates because Fig 3A is shown with a linear y-axis, and hence the exponential phase, if any, is impossible to identify.

To analyse the growth rate, we log-transformed the OD values (black scatterplot) to find the linear region in log-transformed (blue scatterplot), see the Figure below. As mentioned above, we uploaded the script to GitHub: <https://github.com/Knorre-lab/GrowthCurves>.

**Figure Answer to Reviewer Question 1.** An example output of the script applied to each growth curve that was used to compare growth rates of the *rho^−^* and *rho^+^* strains in Figure 3B.

F) The model is very sparsely described to the extent that I cannot follow the reasoning. As I understand it, cells are treated as individual agents, but mtDNA molecules are not, but their copy number is a property of cells?

Yes, correct. The individual cell object is defined as an entity that can harbor a predefined number of mtDNA molecules, has an individual *grande* or *petite* phenotype tag, and includes the parameter “age”, which indicates the time spent since the previous division. We have updated the Methods section with this clarification.

I am left in the dark about the assumptions the model is based on and the settings of parameters and variables. Aging is referred to but it is not clear whether that is replicative or chronological aging, or what distribution age follows, and it is not clear what phenotypes vary with age.

In our model the age is neither replicative nor chronological aging, but the time spent by the cell after the previous mitotic division. We agree that this could be confusing and rephrased the text to avoid this term.

The phenotypes of cells are not explicitly defined or referred to. I am left in the dark about important parameters, such as population size, mutation and recombination rates for mtDNA and nuclear genomes, mating, cell lag times, between cell variability, and any distinction between circular, linear or tandem arrangements of mtDNA.

In the simulation, we keep everything as simple as possible. We did not consider *de novo* mutations and recombinations (though Although the model can be expanded with such parameters, at this stage of the model development we omitted these parameters for the sake of simplicity). We also did not model mating but started simulations with the heteroplasmic cells. The step-by-step model iterations are explained in the Methods section as well as in Figure S7. We updated the Methods section with the population size (10^3^) in simulations.

It is unclear whether all cells divide at the same time, and whether cell division times are the same for haploids and diploids. The reasoning and potential shortcomings and strengths of the choices made by the authors in their modelling are not well illuminated and I cannot evaluate which scenarios are well or poorly reflected in the model.

We updated the Methods section explaining the stochastic model with the following paragraph: “An individual cell was defined as an entity which can harbor a predefined number of wild type and mutated mtDNA molecules, has an individual grande or petite phenotype tag and the parameter indicating the time spent after the previous cell division. During each simulation, the total number of mtDNA molecules within a cell remains constant, while the proportion of mutated and wild-type mtDNA can change due to selection and stochastic drift.”.

The nDNA ploidy of the cells appears not to be important for the model output, as it models only mtDNA, although it could affect the average copy number of the mtDNA in the cells. However, the results were robust to mtDNA copy number in the simulation (Figure S10). To make the modelling part more clear, we tried our best to clarify the logic of the model by updating the “Stochastic modelling” Methods section as well as make some minor changes in Figure S7.

2. I am not convinced by how the authors extract and model intercellular fitness. First, the maximum growth rate (depending on how it is defined) may not reflect the average growth rate across the entire growth phase, and may thus not be reliable as a doubling time approximation. It e.g. looks like in Fig 3A rho+ cells have a growth rate advantage in early growth, but not in later growth phases. Moreover, it looks like (hard to tell, unless the non-linearity of OD and population size are taken into account, and data are shown on a log-scale) there are differences in lag-phase and the final cell yield, between rho+, - and 0. Presumably, these may contribute to cell fitness differences, but this does not appear to be mentioned nor considered in models?

The Methods says that cell doubling times, which seem to be the only determinants of cell fitness (although this is not made explicit), are taken from Fig 2 - but there is not information on cell growth in Fig 2. Presumably, the authors mean Fig 3B? However, it is unclear if the shown growth rates are for haploids or diploids, and whether they are applied to haploid or diploids in the simulation, or both. There are often substantial differences in growth between haploids and diploids.

Finally, the relative fitness of rho- in Fig S5 simulations were set to 0.75, and it is not clear why or how this value relates to the doubling time numbers indicated in the Methods. There are ways to convert a doubling time differences into a selection coefficient, with various assumptions, but it’s not clear what has been done here.

We agree with this comment, the relative growth of *petite* vs *grande* cells in diploid and haploid backgrounds might differ. Moreover, the dynamics of growth rate along the crossing experiment may differ and depend on many secondary conditions. Together this limits the connection of the model to our experimental data. To make this explicit, we added the following sentence to the Methods section: “However, it should be noted that the actual growth rates of *grande* and *petite* cells in diploid cells during crossing experiments may differ from these values.”

The “Stochastic modelling” section incorrectly referred to Figure 2 instead of Figure 3B, and we thank the reviewer for pointing it out. We corrected this mistake in the revised version of the manuscript. Moreover, we replaced Figure S5 (Figure S7 in the revision) relative fitness value with the doubling times as was done in the Methods section.

3. The author’s estimates of the true cell fitness of mutated and intact mtDNA are unlikely to be exact (see above). Moreover, true cell fitness may vary between nuclear genotypes and environments. E.g. ref 16 highlight a situation where the effect of mtDNA on cell fitness is very different from the one modelled here. To me, it would seem very informative to know under what cell fitness assumptions mutated mtDNA, with different intracellular fitness advantages and different heteroplasmy levels, replace intact mtDNA in diploid cell populations. I would invite the authors to not treat fitness as a fixed parameter but as a variable in their simulations.

We agree that true cell fitness may vary between nuclear genotypes and environments. We plan to further develop the model by incorporating variations in intracellular fitness and adding features such as de novo mutations and a non-threshold function for mutant mtDNA pathogenicity. However, we prefer to pursue these developments outside the scope of the current study.

Minor comments:

4. When discussing the yeast petite phenotype, it would be informative if the authors explained the mechanistic origin of the slow growth phenotype (PMID 34799698), which could influence interpretations of data.

We added the sentence in the Introduction noting the reduced ability of *petite* cells to synthesise some of the amino acids. Now, the text looks as follows

“This phenotype is defined by slow growth and an inability to utilise non-fermentable carbon sources [19]. The growth rate of petite cells is also reduced because they have only limited capacity to synthesise some amino acids [20].”

5. Line 138 Fig S2 does not support variation from 3.0 copies, but rather log2 = 2.5 and is probably not the right reference for this statement.

Thank you! We mistakenly referred to Figure S2 instead of Table S2, where we report that the median read depth of all wild-type mtDNA reads is only three times higher than the average read depth of nDNA. This demonstrates that median coverage cannot be reliably used to estimate mtDNA copy number, which explains why we decided not to use it. We have corrected the reference in the revised version of the manuscript

6.Line 147: I cannot find a measure of the correlation in either figure or text.

We rephrased the Figure 1D legend and added the correlation coefficient there. Now it looks as follows “(D) *rho^−^* mtDNA genome size does not show significant correlation with mtDNA copy number per nuclear genome (Kendall's rank correlation tau = 0.105, p-value = 0.45);”

7. Line 433: I am not sure why a maximum is used? Presumably, deviations from the true copy number would be equally likely to go in both directions – and a median or average would seem more appropriate?

Our *rho^−^* strains retained different regions of the parental full-length mtDNA, suggesting the use of distinct mtDNA regions for quantifying mtDNA copy number. Sequencing of *rho^−^* strains revealed low coverage in certain regions while maintaining high coverage in others. For instance, Table S2 shows that strains *rho^−^* 5 and *rho^−^* 6 have very low read depth in the second region (8002-8153). We propose that this may arise from a secondary mutation involving the deletion of one region to a sufficient extent, thereby displacing the original *rho^−^* variant. We reasoned that in the case of secondary deletions, max-covered regions would be the most likely to be shared between the majority of mtDNA variants present in the strain population. Consequently, we employed the max function in our pipeline instead of more complex logic involving thresholds.

8. Line178: Are these strains and the method with which they were obtained described?

We added an explanation on how we obtained spontaneous *rho^−^* strains to the Methods section under “Yeast strains, growth conditions and reagents”. As this was a post-hoc analysis, we included the data on additional strains available in our laboratory which were not specifically generated for this study. For the additional strains, we used different parental strains (with a different mating type or selective marker), however, there were no additional alterations to their nuclear DNA, allowing us to combine them for this comparison.

9. Line181: Do the authors have a rough estimate or interval for the number of generations? As is, it is hard to interpret the associated statements.

Unfortunately, we do not have the estimates, though we agree that such analysis could benefit from them. We can assume that the subsequent measurements of suppressivity were done after at least 20 generations (a rough estimate of the number of divisions); however, given that this was not controlled, we prefer not to speculate about this. Furthermore, since Ne was not experimentally controlled in these experiments, we removed the mention of Ne from the figure. Instead, we added an explanation on the process of passing the culture through a single colony and the passages that occurred before and after freezing. We also made a minor adjustment to the figure (removed the arrows that could cause confusion).

## Reviewer #2:

In their article titled „Inheritance bias of deletion-harbouring mtDNA in yeast: the role of copy number and intracellular selection” Kashko et al. investigate the phenomenon known as mtDNA suppressivity in which mtDNA molecules harbouring deletions (rho-) compete with or even entirely outcompete intact mtDNA. The study examines two factors contributing to suppressivity: higher mtDNA copy numbers in cells with deletions and a competitive advantage of these deletion-containing mtDNAs within cells. The study uses experimental data from 22 yeast strains and a computational model to determine that both factors play significant roles. The findings suggest that mtDNA deletions can have a selective advantage within the cell, even if detrimental to the organism. The study also explores the influence of genetic bottlenecks on mtDNA suppressivity and discusses potential mechanisms driving this intracellular selection.

The study is interesting, because it provides insight into the broader question how cells maintain the integrity of their mtDNA. The phenomenon that mutant mtDNA’s can outcompete intact mtDNA has been known for a while, and it has been discussed that this is due to a replicative advantage of rho- due to their shorter length or higher density of replication origins. This study sheds further light on the phenomenon of mtDNA suppressivity and provides an interesting stochastic model that helps to understand the mecnahisms underlying suppressivity. While interesting, I think the study would benefit from a slightly more detailed analysis of the sequence features in the rho- genomes that are responsible for the suppressivity.

Points to be addressed:
1. The sequencing of the rho- genomes is a great resource. Figure 1B is nice, but it is difficult to evaluate what features (origins of replication) may correlate with suppressiveness. Perhaps it would be better to depict this figure in a linear format and sort the sequences based on the starting point. The authors state that they 'did not find a strong association between the retained replication origins and suppressivity', a figure detailing this statement would be helpful.

To answer this question, we extended the origin location coordinates up to the middle of the circular map. In the revised version, Figure 1B appears as follows.

**Figure Answer to Reviewer Question 2.**

The authors state that 'two low-suppressive strains, namely IIa10 and 13, lack all three active replication origins: ORI2, ORI3, and ORI5'. How are such genomes replicated.

These origins are more active than the others; this does not exclude the possibility that other origins enable the maintenance of mtDNA. In one of the previous studies, it was shown that *rho^−^* mtDNAs are maintained with other origins or, in rare cases, without intact replication origins (DOI: 10.1038/292075a0). We updated the introduction with this reference and the following text:

“Such mutants typically contain large deletions in their mtDNA sequences but usually retain at least one of the eight replication origins [15–17]. Some of these replication origins, namely ORI2, ORI3 and ORI5, are reported to be more active than others [18].”

2. The high copy number of mtDNA in some rho- strains is astonishing. According to the graph in figure 1D two strains, one of which has a 50kB mtDNA, have a mtDNA copy number of approximately 2^9=512. To validate these results, but also to characterize the effects on the mitochondrial network, the authors should perform a DAPI staining of the cells and perform quantitative fluorescence microscopy.

To answer this question, we chose three strains with presumably high (*rho^−^* 5, *rho^−^* 20, *rho^−^* 11) and low (*rho^−^* 13, *rho^−^* 21, *rho^−^* 10) integral mtDNA length (copy number X genome size) and stained them with DAPI. For this, cells were fixed in 70% ethanol and incubated with DAPI at a final concentration of 2 µg/mL for 30 minutes, then resuspended in 1x PBS and analysed under Olympus BX51 microscope with the following filter set: U-MNU2 filter for DAPI (excitation λ =360 nm – 370 nm; dichroic mirror λ =400 nm; emission wavelength > 420 nm), same exposure, same image processing in GIMP. We also used the parental *rho^+^* strain as a control.

As shown in Figure [Answer to Reviewer Question 3], in some *rho^−^* strains (e.g., *rho^−^* 5), we observed high-intensity fluorescence not connected to the nucleus. We assume that the largest DAPI-positive area corresponds to the nucleus, while smaller foci correspond to mitochondrial nucleoids. The figure illustrates that some *rho^−^* strains may have an extremely high total extranuclear mtDNA content. However, the resolution of the microscopes available to us did not allow us to distinguish between mtDNA attached to the nucleus and the nucleus itself. Therefore, we did not pursue this line of experiments, although we agree that rho− variants with exceptionally high total mtDNA length are of interest and could exhibit unusual properties that could be assessed using fluorescent microscopy.**Figure Answer to Reviewer Question 3**. DAPI staining of *rho^−^* and *rho^+^* cells. The arrows point to a bright focus that is not connected to the cell nucleus (the largest bright spot).

It should also be noted that, given the likely presence of secondary mutations in the total mtDNA pool of a *rho^−^* strain, we might be overestimating the integral mtDNA amount by accounting only for the original deletion. In other words, while we assume the original deletion coordinates obtained from NGS to plot Figure 1D, the actual mtDNA content per cell might be lower due to the accumulation of shorter mitochondrial genomes with secondary deletions, which was not controlled for in our experiments. We added the following text to the Discussion to explain this possibility:

“We did not find a positive correlation between genome size and either mtDNA copy number or suppressivity in the rho− strains (Figure 1D, Figure S4). On the one hand, the absence of such correlation could be explained by the absence of contribution of the sequence length to the rho− mtDNA variant suppressivity. On the other hand, it may also result from the presence of mtDNAs with secondary deletions in the *rho^−^* strains. Such mtDNAs if accumulated in high copy numbers can strongly affect suppressivity.

3. For better assessment of the data, it would be helpful to include heatmaps in figures C, D and E (similar to Fig S2) that display the mtDNA genome length (for C), the suppressivity (for D) and the mtDNA genome length (for E).

We updated the figures with the heatmaps as suggested.

It would also be helpful to label the dots that represent rho- genomes that are used in figure 2. Perhaps that could also be done in a supplementary figure.

To avoid overloading the figure with labels, we included a copy of Figure 1C with all strains labeled in the supplement as Figure S3.

4. It is very interesting to see in Figure 2 that the suppressivity changes in subclones. However, the molecular reasons for that remain unknown. It would be very interesting to sequence the subclones to determine, whether secondary mutations within the mtDNA, as the authors discuss, are found. I feel that such an experiment would strengthen the molecular insight of the story.

To address this question, we sequenced two sets of subclones from Figure 2 and added panel B to present the results. Our analysis revealed that all five studied subclones of the *rho^−^* strain IIc11 contained secondary deletions, even though not all tested subclones showed changes in suppressivity. Furthermore, for Ia14, we identified one subclone with a major secondary deletion, two with minor deletions, and two that retained full-length parental mtDNA variants. We updated the Results section and the Figure 2 legend to include these findings and expanded the discussion by adding several sentences to explain that six out of ten tested subclones exhibited pronounced secondary deletions.

5. The stochastic model is interesting. However, I have problems understanding how the mtDNA copy number was integrated into the model. The determination of the mtDNA copy number in the rho- strains indicates greatly varying numbers. In Figure S5 it is stated that both of the parental cells contribute 10 mtDNA copies into the zygote. How are these numbers linked to the 'Heteroplasmy level (rho-)' (x-axis Figure 4A)? More information on how the simulation was set up would be helpful for the reader to interpret the findings.

In the revised version of the manuscript we updated the “Stochastic modelling” section in Methods as well as Figure S7. We have clarified that during each simulation, the total number of mtDNA molecules within a cell remains constant, while the proportion of mutated and wild-type mtDNA can change due to selection and stochastic drift.

I am somewhat confused about the 'Intracellular rho- mtDNA fitness' value. In the text it is stated that it is within 0 and 3. In Figure 4A it is between 0 and 4 and in Figure 4B it is between 0 and 2. What is the reasoning behind this?

Thank you! In the simulation, we imputed the intracellular fitness levels in the range between 0 and 4. We corrected the corresponding text.

As it can be seen in Figure 4A, intracellular fitness values within the range of 2 to 4 produced similar results for most initial heteroplasmy levels. Therefore, for further analysis, we only used the data obtained from simulations with intracellular fitness values ranging from 0 to 2. We updated the Results text by mentioning this step.

Other points:

1. I find Figures 3C and 3D difficult to follow. More information should be given in the text about the experiment or the figure. What strains carry the URA+-marker? How long are the cells incubated? What was the expectation?

In this experiment, we intended to assess the growth rates under the conditions of the suppressivity measurement experiment. For this, we quantified CFUs for parental strains and their diploid progeny at the beginning of the mating experiment (0 h) and at the end (20 h) and used the ratio between CFUs at the last vs first timepoints as the growth rate approximation. The parental strain carrying the *URA^+^* marker is indicated on the X axis of the plot (for Figures 3D and 3E), while the second parent was *LEU^+^ ura^−^ rho^+^* strain in all cases.

We updated the Methods section with more details on this experiment, which can now be found under “Growth rate assessment”. We also updated the scheme (Figure 3C) and some labels on Figure 3D and 3E to make the data easier to follow.

2. What is the difference between 'Suppressiveness' and 'Suppressivity' in Fig S3? I assume it is the same, but then it seems unnecessary to include a heatmap

Yes, this is the same. We agree, the heatmap was removed. Thank you!

3. What is Ne in Figure S4?

In Figure S4 (now Figure S6), the notation "Ne" initially represented either passing the culture through a single colony (Ne = 1) or broader bottlenecks during culture passages. However, since Ne was not experimentally controlled in these experiments, we removed the mention of Ne from the figure. Instead, we added an explanation detailing the process of passing the culture through a single colony and the passages that occurred before and after freezing. We also made some minor adjustments in the figure (removed the arrows that could be confusing)

4. Line 139: Some context should be given to why the results of 3-15-5 copies of mtDNA per cell is unrealistic.

We removed the word "unrealistic" because it could be regarded as subjective and evaluative. We updated the next sentence by adding references to other studies where authors have measured mtDNA copy numbers. Now the text looks as follows “For instance, according to NGS, the mtDNA copy number of the control wild-type strain ranged from an unrealistic 3.0 to 15.5 copies per haploid nuclear genome, depending on the mtDNA region selected as reference (Table S2). In contrast, qPCR provided a higher estimate of 22.2-26.6 mtDNA copies per nuclear genome (Table S4), which aligns better with the estimates reported in other studies [27–29].”

5. Line 172: What is meant by 'convergent'?

By ‘convergent’ we meant values that were comparable, reproducible, and similar. To avoid further confusion, we replaced "convergent" with "similar" in the text. Now the sentence reads as follows: “At the same time, some subclones obtained from single cells showed similar suppressivity, which, however, strongly differed from the average suppressivity of the parental *rho^−^* strain.”

6. Line 178: A little more context to the 76 strains would be necessary. I assume those are rho- strains, but this should be stated.

Yes, these were indeed *rho^−^* strains, we added this information where we first introduced the strains. As this was a post-hoc analysis, we included the data on additional strains available in our laboratory which were not specifically generated for this study. The way of making was the same for all strains. Namely, they arose due to spontaneous mutations in the mtDNA of a parental strain and were selected on YPDGly medium as single colonies having a *petite* phenotype. We used different parental strains (with a different mating type or selective marker). However, there were no additional alterations to their nuclear DNA, allowing us to combine them for this comparison.

We clarified this in the main text by adding the following sentence: “These strains include the *rho^−^* strains sequenced in this study, as well as additional spontaneous *rho^−^* strains for which multiple suppressivity assessments were available. They include other spontaneous *rho^−^* strains originated from the same W303 *URA^+^ mat alpha* parental *rho^+^* strain which were not selected for sequencing, and *rho^−^* strains obtained from different parental strains with the W303 background not carrying additional mutations in their genomic DNA”.

7. Lines 292-294: The authors speculate that the increase of mtDNA in rho- cells is due to a compensation mechanism. Why do the copy numbers vary so drastically between the strains? The authors could comment on this.

We propose that the primary explanation lies in *rho^−^* mtDNA copy numbers being subject to less regulation, including a negative feedback loop mediated by mitochondria-encoded proteins. This lack of regulation allows mtDNA copy numbers to increase and drift stochastically, resulting in significant heterogeneity in mtDNA levels across cells and strains. Concurrently, certain replication origins may exhibit higher activity than others, potentially driving elevated mtDNA copy numbers per cell in *rho^−^* strains.

We have revised the text to incorporate these hypotheses. The updated paragraph addressing the regulation of *rho^−^* mtDNA copy numbers now reads as follows:

“To unravel how the mtDNA copy number in wild-type and *rho^−^* *S. cerevisiae* cells affects their inheritance bias, we studied several rho− strains harbouring large deletions in their mitochondrial genomes. mtDNA copy number per cell in all studied rho− haploid strains exceeded that of the parental *rho^+^* strain (Figure 1C). This observation is concordant with a previous study showing that *rho^−^* yeast cells accumulated mtDNA to a copy number ~80 times higher than that in a wild-type strain [24]. Furthermore, we have recently found that the total amount of mtDNA is higher in *rho^−^* cells, whereas in some strains, the average mtDNA amount exceeded that of *rho^+^* cells several fold [38]. We speculate that a negative feedback loop mechanism induces mtDNA biogenesis to compensate for a deficiency in mitochondrial function that eventually leads to excessive accumulation of mtDNA. A similar mechanism was described in nematodes, where the mitochondrial unfolded protein response transcription factor ATFS-1 helped to maintain high levels of mtDNA variant harbouring a deletion by enhancing the binding of mtDNA polymerase to mutated mtDNA molecules [39]. To the best of our knowledge, such a mechanism is still unknown in yeast. Meanwhile, deletion of the COX5 gene, encoding an essential component of cytochrome oxidase, the terminal part of the respiratory chain, increases mtDNA copy number per cell [40]. Furthermore, we have recently shown that *rho^−^* cells are usually more heterogeneous by the level of mtDNA than their parental *rho^+^* cells [38]. Together, these observations suggest that the ability to encode essential components of OxPhos is necessary for maintaining constant mtDNA levels across generations. If this is true, rho− cells lacking such regulation would experience drift in mtDNA copy number, resulting in highly heterogeneous mtDNA copy numbers both per cell and per clone. This hypothesis is consistent with the previous observation that *rho^−^* cell suspensions are more heterogeneous in terms of mtDNA levels per cell compared to their parental *rho^+^* strains [38]. Moreover, this hypothesis is supported by the observed variability in mtDNA copy number among rho− strains in this study (Figure 1C-E). However, further studies are necessary to elucidate the properties and mechanisms underlying the regulatory processes linking OxPhos and mtDNA maintenance.”

8. Line 299: There are many deletions that lead to compromised OXPHOS. Is the deletion of COX5 the only one that leads to more mtDNA?

To the best of our knowledge, this question remains understudied, and we cannot provide a definitive answer at this stage. One reason for this is that compromised OXPHOS can influence mtDNA copy numbers, while the inactivation of OXPHOS also removes the necessary selection pressure for mtDNA maintenance, leading some cells (subclones) to eventually lose their mtDNA. This complexity makes interpretation challenging. Therefore, we prefer not to over-speculate on this topic, although we believe it is worthy of further investigation.

## Reviewer #3:

The manuscript by Kashko and collaborators argues for a model in which rho- mitotypes become dominant in heteroplasmic diploid cells due to increased copy number and preferential duplication/selection during growth. The current manuscript is quite interesting but the main arguments would be improved by some additional analyses.

A. The author’s model for the mechanism of suppression by rho- variants is that the rho- mtDNA is replicated/inherited more readily and can displace the rho+ mtDNA within a cell lineage. This model has several testable hypotheses that have not been addressed in the current manuscript but should be.

1. In a cross between a suppressive rho- strain and a rho+ strain, the rho- diploids should have mtDNA that matches the rho- strain, which can be easily determined using NGS.

To answer this question and verify that petite diploid strains obtained via the crossing of *rho+* and *rho⁻*  variant harbour the same *rho⁻* variant, we designed primers specific to *HS rho⁻* mtDNA, which are complementary to yeast mtDNA sites near the deletion and directed toward the deletion (30525R–32063F).

primer 1 mtDNA32063F: TGATAAAAATTAAGATGAAGACT;

primer 2 mtDNA30525R: CCCCAGATATCTTCTTGTTA

PCR using this primer pair with mtDNA from the HS *rho⁻* strain is expected to produce a 0.7 kb product, which we confirmed by using genomic DNA extracted from the HS *rho⁻* cells as the template. No expected PCR product was detected with these primers when total DNA from the *rho^+^* or *rho⁰* strain was used as the template (see Figure Answer to Reviewer Question 4). At the same time, we were able to recover the PCR product of the expected size from DNA isolated from three *petite* strains generated by crossing *rho^+^* with *HS* *rho⁻* (Figure Answer to Reviewer Question 4).

.

**Figure Answer to Reviewer Question 4.** PCR products amplified using primers 30525R and 32063F with the following template DNA: lanes 1, 2, and 3 represent *petite* colonies derived from crosses between the *HS rho⁻* and *rho⁺* strains; *rho⁺* represents the control *rho⁺* strain; *rho⁻* represents the control *HS* *rho⁻* strain; *rho⁰* represents the control *rho⁰* strain.

Furthermore, as demonstrated (see Figure 2B of the revised manuscript), passing the strain through the stage of the individual colony results in the fixation of secondary mutations in mtDNA. A similar process may occur during crossing experiments, such that some diploid strains could already harbor mtDNAs with secondary mutations or, given that recombination between *rho⁺* and *rho⁻* mtDNA is theoretically possible, recombinant variants.

Consequently, we contend that our PCR-based experiment provides a qualitative assessment of the relevance of our model. However, further exploration of this topic requires extensive additional studies, which extend beyond the scope of the present manuscript.

2. In a cross between a suppressive rho- strain and a rho+ strain, the rho+ diploids should be heteroplasmic intermediates, and both rho- and rho+ mtDNA should be observed in the NGS.

To answer this question and test whether *rho⁻* mtDNA can be retained in the diploid strain in a state of heteroplasmy, we performed an additional experiment in which we crossed a *rho⁺* yeast strain with an *HS rho⁻* strain harboring a mutated mitochondrial genome. We then analysed whether the diploid strain retained the *HS rho⁻* mtDNA using PCR with primers designed to identify *HS* *rho⁻* mtDNA (see the answer to question 1)

Using the *HS rho⁻-*specific primers, we demonstrated that *rho⁻* mtDNA persisted in the resulting diploid cell lines for multiple generations. In seven out of ten cells that underwent at least 20 divisions after crossing, we detected *rho⁻* mtDNA via PCR.

Figure Answer to Reviewer 5. PCR reveals *HS rho⁻* mtDNA in diploid strains generated by crossing the BY4741 *mat a rho⁺ TRP1* strain (labelled as BY a TRP1 №1823) with the *HS rho⁻* strain (petite3α URA3 №681).

B. The paper would be greatly strengthened by more detailed analysis of the variability of suppressivity observed after genetic bottlenecks. The authors claim (lines 173-174) that their results are suggestive of additional mtDNA mutations in rho- strains. The authors should do NGS on the subclones with large changes suppressiity (e.g. Rho- la14, Rho- 2, and Rho- 21, or the equivalent). Are their additional mutations? Or do changes in suppressivity correspond to subclones with increased/decreased rho- mtDNA copy numbers, which would be more in line with the authors’ model.

To address this question, we sequenced two sets of subclones from Figure 2 and added panel B to present the results. Our analysis revealed that all five studied subclones of the *rho^−^* strain IIc11 contained secondary deletions, even though not all tested subclones showed changes in suppressivity. Furthermore, for Ia14, we identified one subclone with a major secondary deletion, two with minor deletions, and two that retained full-length parental mtDNA variants. We updated the results section and the Figure 2 legend to include these findings and expanded the Discussion by adding several sentences to explain that six out of ten tested subclones exhibited pronounced secondary deletions.

C. I think Fig 5B is misleading in the context of the data for this paper. This figure attempts to illustrate the authors’ suggestion of a “ratchet” in which more suppressive mtDNA variants will overtake a rho- cell population. However, the authors have not performed this experiment (though they could). Note that this mechanism requires that (1) suppressive mtDNA variants are constantly arising (not demonstrated in this manuscript) or (2) there is a selective advantage for suppressive mtDNA variants (which is in contrast to the authors’ data). A much better Fig 5B that would illustrate the gradual loss of the rho+ mtDNA within a heteroplasmic population with a suppressive rho- mtDNA and would serve to explain why rho+ cells dominate in normal populations (Fig 5A) but why rho- diploids are recovered with suppressive heteroplasmic mtDNAs.

We agree that Figure 5B is somewhat speculative; however, in our opinion, it provides a likely explanation of the genetic processes occurring in *rho^−^* cell suspensions (populations), where there is no cell-level selection and more selfish mtDNA variants repeatedly emerge within the cells. This figure is intended as a hypothetical scheme, and we acknowledge that we cannot definitively prove all the processes depicted. Nevertheless, our study (including the experiments added after the revision) supports several key observations: (1) Secondary mutations in *rho^−^* mtDNA are often and easily fixed (Figure 2B). (2) *rho^−^* variants exhibit significant variation in intracellular fitness (Figure 4C). (3) There is a gradual increase in suppressivity among *rho^−^* strains (Figure S6).

To clarify our speculations, we have rewritten the paragraph explaining Figure 5B to improve its clarity. The revised text now reads as follows:

“In line with our results indicating that some *rho^−^* mtDNAs can have higher intracellular fitness than others, we have shown that the suppressivity of yeast *rho^−^* clones can be changed after the genetic bottleneck (Figure 2) and, on average, increase with generations (Figure S6). We showed that passing a *rho^−^* strain through a single colony fixes secondary mutations in its mtDNA that can contribute to the increase in its suppressivity (Figure 2B). The gradual increase in suppressivity could be attributed to two factors. On the one hand, cells with secondary mutations in mtDNA leading to a higher suppressivity proliferate faster than parental cells with the original mutation. However, Figure 3 shows that there is no pronounced difference between the growth rate of *rho^−^* strains with different deletions. On the other hand, spontaneous secondary deletions in rho− mtDNA could lead to the appearance of mtDNA variants with intracellular fitness exceeding the intracellular fitness of the original *rho^−^* mitotype. Several experiments in our study support the second possibility. First, different rho− mtDNA molecules can have various intracellular fitness (Figure 4C). Therefore, *rho^−^* cells will eventually produce new *rho^−^* variants with increased intracellular fitness, as evidenced by the post hoc analysis in Figure S6. Due to an increase in intracellular fitness, this variant is expected to displace parental *rho^−^* variants within the same cell and all descendants of this cell. In the presence of *rho^+^* cells, such variants are expected to be removed from the population because of the selection on the cell level (see a hypothetical scheme in Figure 5A). Meanwhile, in petite cell populations, newly emerging mtDNA variants with low intracellular fitness are expected to be outcompeted and removed from the population, while the mtDNA with high intracellular fitness will repeatedly emerge and displace the parental variants within cell lineages (Figure 5B). We propose that as a result, a ratchet-like mechanism leads to a gradual increase in the proportion of cells carrying suppressive *rho^−^* mtDNA within small yeast populations. However, this process should be limited by the maximal possible intracellular fitness of the *rho^−^* mtDNA.”

Minor comments.

How many nuclear genome mutations are recovered in the spontaneous rho- strains? Do any affect genes involved in mitochondrial maintenance and aerobic respiration?

Given that the mutation rate of nDNA is quite low compared to the frequency of deletions in mtDNA, and the read depth of nDNA was relatively low in our study, we did not attempt to recover nDNA mutations. Furthermore, it could be expected that most nDNA mutations are recessive and would not affect the *petite*/*grande* phenotype in the heteroplasmic state. We also previously tested that the *HS rho^−^* strain has no nDNA mutations affecting suppressivity: the complete depletion of mtDNA from this *rho^−^* strain makes it *rho^0^*, which exhibits a background suppressivity level of ~5% (Karavaeva et al., 2017, PMID: 28193734 / [doi.org/10.1242/jcs.197269](http://doi.org/10.1242/jcs.197269) and not shown).

Nonetheless, considering that previous papers report an increase in the mutation rate in yeast *petite* strains (doi: 10.1093/genetics/83.4.645; doi: 10.1534/g3.113.010108), we agree that this question is worth further investigation; however, we believe it appears to be slightly out of the scope of this study.

Fig 1B would be more interpretable if the rho- genomes were sorted in some way (e.g. by suppressivity?) For example, it appears that the most suppressive mitotypes include both ORI2 and ORI7 in contrast to the authors’ assertion (lines 114-117) that there weas no association between replication origins and supressivity.

The *rho^−^* genomes were sorted according to their size (larger genomes are close to the center). We updated the legend of Figure 1 to mention this. Moreover, to make the figure more accessible, we extended the coordinates of the ORIs to the center of the circle (see the updated Figure 1B).

To improve comparison with previous studies that have used the mtDNA copy number from 14,000-20,000, the authors should define a correction factor in the rho+ strain (and potentially other strains) that allows NGS-based copy numbers from their three chosen regions to be transformed into a 14,000-20,000 NGS copy number measurement. Does this correct the differences in mtDNA/nDNA ratios between NGS and qPCR methods?

Indeed, the correction coefficient calculated from *rho^+^* read depth coverage partially explains the difference between qPCR and NGS estimation. We updated the results section with the following sentence: “Notably, these regions had the read depth 2.3-3.4 fold lower than the read depth of the GC-rich 14,000-20,000 region, suggesting that the actual mtDNA copy number estimate using these regions could be underestimated by several fold (Table S2).”

The axes in Fig 1D should be switched for consistency between Figs 1C-1E.

Done.

The authors should add a panel to Fig 1 displaying suppressivity as a function of genome length.

We (1) incorporated the heatmap illustrating suppressivity into Figures 1D and 1E, and (2) included the aforementioned scatter plot as Figure S4.
